# Laparoscopic hepatectomy for the treatment of hepatic alveolar echinococcosis

**DOI:** 10.1051/parasite/2021001

**Published:** 2021-01-13

**Authors:** Li Wan, Bo Ran, Tuerganaili Aji, Paizula Shalayiadang, Tiemin Jiang, Yingmei Shao, Hao Wen

**Affiliations:** 1 Department of Hepatobiliary & Hydatid, Digestive and Vascular Surgery Centre, First Affiliated Hospital of Xinjiang Medical University 830011 Urumqi PR China; 2 State Key Laboratory of Pathogenesis, Prevention and Treatment of High Incidence Diseases in Central Asia, Xinjiang Medical University 830011 Urumqi PR China; 3 Xinjiang Hydatid & Hepatobiliary Surgery Medical Centre 830054 Urumqi PR China

**Keywords:** Alveolar echinococcosis, Laparoscopic hepatectomy, Minimally invasive

## Abstract

*Background*: At present, laparoscopy is relatively mature as a minimally invasive technique, but there are few reports on this approach for the radical treatment of hepatic alveolar echinococcosis (AE). In this study, we aimed to evaluate the safety and feasibility of laparoscopic hepatectomy (LH) for AE treatment. *Results*: A retrospective review of medical records obtained from 13 patients diagnosed with AE between January 2018 and December 2019 and treated with laparoscopic hepatectomy was conducted at the First Affiliated Hospital of Xinjiang Medical University. All patients (*n* = 13) underwent hepatic resection using laparoscopy and none were transferred to open surgery. The average duration of surgery was 285 min (145–580 min). Intraoperative bleeding was 305 mL (20–2000 mL). The mean duration of postoperative catheterization was 6.9 days (3–21 days), and postoperative hospital stay was 7.2 days (4–14 days). No complication of Clavien-Dindo grade III or above occurred, except for the second patient with acute liver failure post-surgically. No recurrences or deaths were observed at 9–30 months of follow-up. *Conclusions*: Laparoscopic hepatectomy appears to be safe and effective in selected AE patients. The advantages of this technique for AE treatment need to be further compared with the classical open approach.

## Abstract (fr)

*Contexte :* À l’heure actuelle, la laparoscopie est relativement mature en tant que technique minimalement invasive, mais il existe peu de rapports sur cette approche pour le traitement radical de l’échinococcose alvéolaire hépatique (EA). Dans cette étude, nous avons cherché à évaluer la sécurité et la faisabilité de l’hépatectomie laparoscopique pour le traitement des EA. *Résultats :* Une revue rétrospective des dossiers médicaux obtenus auprès de 13 patients diagnostiqués pour EA entre janvier 2018 et décembre 2019 et traités par hépatectomie laparoscopique a été menée dans le premier hôpital affilié de l’Université médicale du Xinjiang. Tous les patients (*n* = 13) ont subi une résection hépatique par laparoscopie et aucun n’a été transféré en chirurgie ouverte. La durée moyenne de la chirurgie était de 285 min (145 à 580 min). Le saignement peropératoire était de 305 ml (20 à 2 000 ml). La durée moyenne du cathétérisme postopératoire était de 6,9 jours (3 à 21 jours) et l’hospitalisation postopératoire était de 7,2 jours (4 à 14 jours). Aucune complication de grade III ou supérieur (Clavien-Dindo) n’est survenue, sauf pour le deuxième patient présentant une insuffisance hépatique aiguë après la chirurgie. Aucune récidive ou décès n’a été observé à 9-30 mois de suivi. *Conclusions :* L’hépatectomie laparoscopique semble sûre et efficace chez certains patients atteints d’EA. Les avantages de cette technique pour le traitement des EA doivent être mieux comparés à l’approche ouverte classique.

## Introduction

Hepatic alveolar echinococcosis (AE) is a chronic proliferative parasitic disease, with a poor natural prognosis due to its characteristic infiltrative growth [15]. It is reported that mortality in untreated or inadequately treated AE patients can be as high as 90% after 10–15 years of diagnosis [1, 8, 17]. Therefore, the expert consensus is that patients with diagnosed AE should be given timely surgery or drug treatment [4, 5]. To date, radical surgical resection has still been considered the main method of AE treatment, despite the increased interest in nonsurgical techniques [1–3, 7, 9, 11–14, 16]. In recent years, laparoscopic hepatectomy (LH) has been well accepted by patients since it is minimally invasive. Therefore, LH has been proposed for the treatment of AE and is used in clinical practice.

In this retrospective analysis, 13 AE cases admitted to our centre from January 2018 to December 2019 were included to discuss the treatment outcomes of LH and propose initial possibly selective criteria.

## Patients and methods

### Ethics statement

All patients in this study provided written informed consent before surgery. This study was approved by the Ethics Committee of the First Affiliated Hospital of Xinjiang Medical University (No. 20200116-04).

### Patients

A total of 13 patients (male: 6, female: 7) receiving LH were enrolled in this retrospective analysis. Eligible patients were those confirmed with AE lesions located within the half-liver by using colour doppler ultrasound (US) and computed tomography (CT). In addition, the liver remnant was adequate (≥40%). Finally, patients could tolerate a laparoscopic operation on the basis of their cardiac and pulmonary function. The exclusion criteria were as follows: patients’ lesions have invaded important vessels of the hilum and/or patients have developed extra-hepatic distant metastasis.

### Operation

The patients were in the supine position. Routine disinfection and draping were performed after the onset of anaesthesia. Next, a small incision of about 1.2 cm was made above the umbilicus and a Veress needle was placed. Subsequently, CO_2_ pneumoperitoneum was established, and intra-abdominal pressure was maintained in a range of 10–12 mmHg. Afterwards, an inspection hole was made at a position that was above the navel in order to introduce the laparoscope. On this basis, the lesion size, location and adhesion to the surrounding tissues were evaluated. Once the characteristics of lesions were clarified, the remaining four trocars were successively entered under the visualisation of a monitor. Operative procedures followed the steps described below. Step 1: the first hepatic portal was adequately separated from the peripheral tissues. Step 2: the diseased lobe was adequately separated from the peripheral tissues and organs by ultrasound scalpel. Step 3: the pre-cut liver line was marked on the liver tissue 1–2 cm away from the lesion with the coagulation hook. Step 4: after blocking the first hepatic portal vessels, the diseased lobe was resected using an ultrasound scalpel along the pre-cut liver line. In the presence of significant capillary haemorrhage, immediate haemostasis was carried out using a mono-polar electrocoagulation or ultrasound scalpel. Meanwhile, large blood vessels and bile ducts were closed using absorbable clips until total diseased liver was completely resected. Step 5: monopolar electrocoagulation was used for electrocauterisation of the hepatic wound surface. Re-examination was carried out to observe whether active bleeding and/or biliary fistula were present. Local surgical areas were covered using biological absorbable haemostatic gauzes. Step 6: the diseased liver lobe was placed into a sampling bag, and then the bag was extracted out from the abdominal wall through the extending subxiphoid incision. Step 7: a drainage tube was inserted around the surgical region.

## Results

### Patient characteristics

**Figure 1:**
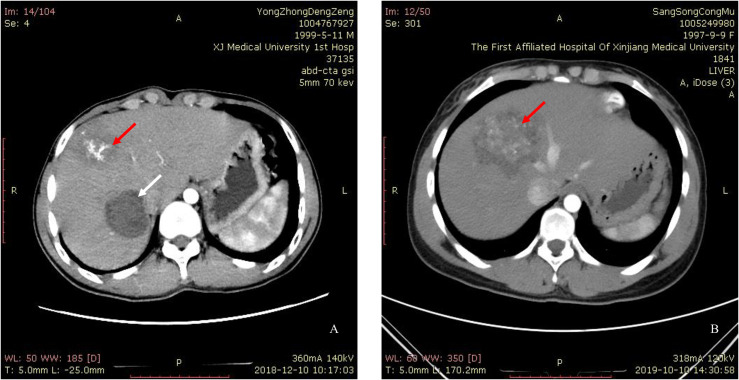
(A) An AE lesion located in the right anterior lobe of the liver (red arrow) and a CE cyst located in the right posterior lobe of the liver (white arrow). (B) The AE lesion is located in the middle liver lobe (red arrow).

The characteristics of the 13 patients enrolled in our study are summarized in Table 1. According to the WHO-IWGE classification criteria, all 13 patients in our study belong to P1N0M0/P2N0M0 stages of hepatic AE. The mean age was 32.7 years (18–52 years). The mean BMI was 22.8 kg/m^2^ (18–33 kg/m^2^). None of the patients had clinical discomfort when they sought medical attention, except two patients who had dull pain in the right upper abdomen preoperatively. The serology results of all patients were positive, and the residual liver volume were adequate (>40%) by preoperative imaging assessment. No patient had received albendazole treatment before surgery. Of note, the fifth patient was also found to have a CE cyst on the right liver lobe in addition to an AE lesion, and the AE lesion of the eleventh patient was located within the middle liver lobe (Fig. 1).

**Table 1:** Baseline information for 13 patients.

Case	Sex	Age	BMI	Child-Pugh	ABZ	Liver lesion				Serology	PHOAS	Sym	Other disease
						PNM	Loc	Size	Num				
1	M	18	20	A	No	P1N0M0	RPL	8 × 5	1	+	No	No	No
2	F	52	26	A	No	P2N0M0	RL	11 × 8	1	+	Yes	No	Hypertension
3	M	33	21	A	No	P1N0M0	LLL	14 × 9	1	+	No	No	No
4	F	42	22	A	No	P2N0M0	RL	9 × 7	1	+	No	No	No
5	M	19	19	A	No	P1N0M0	RAL	4 × 4	1	+	No	No	No
6	M	36	23	A	No	P2N0M0	RL	11 × 9	1	+	No	No	No
7	F	22	22	A	No	P1N0M0	RPL	5 × 5	1	+	No	No	No
8	M	30	18	A	No	P1N0M0	LLL	3 × 2	1	+	No	No	No
9	F	38	26	A	No	P2N0M0	LL	7 × 5	1	+	No	Dull pain	No
10	F	33	19	A	No	P1N0M0	RAL	4 × 3	1	+	No	No	No
11	F	22	22	A	No	P2N0M0	ML	8 × 7	1	+	No	Dull pain	No
12	M	28	33	A	No	P1N0M0	LLL	10 × 8	1	+	No	No	No
13	F	52	26	A	No	P1N0M0	LML	8 × 4	1	+	Yes	No	No

RPL: right posterior lobe; RL: right lobe; LLL: left lateral lobe; RAL: right anterior lobe; ML: middle lobe; LL: left lobe; LML: left medial lobe. PHOAS: pre-operation history of abdominal surgery. PNM (WHO/IWGE [4]): P1: peripheral lesion without proximal vascular and/or biliary involvement; P2: central lesions with proximal vascular and/or biliary involvement of one lobe; N0: No regional involvement; M0: No metastasis. BMI: body mass index (kg/m^2^). ABZ: albendazole; Loc: location; Num: number; Sym: symptoms.

### Intraoperative conditions

LH was successfully completed in all patients. The average operation duration was 285 min (145–580 min), and the average blood loss was 305 mL (20–2000 mL). Two patients were given blood transfusion due to excessive intraoperative bleeding and a long operating time. Seven patients has simultaneous laparoscopic cholecystectomy (LC) due to concurrent chronic cholecystitis. Interestingly, pathology examination results confirmed that the lesion was AE and there was no residual lesion at the resection margin (Table 2).

**Table 2:** Operative parameters for 13 patients.

Case	Date of operation (yyyy/mm/dd)	Operation				Details
		Duration (min)	Bleeding (mL)	Blood transfusion (units)	Residual lesions	
1	2018.02.11	375	200	0	NO	LWR + LC
2	2018.05.10	400	2000	3	NO	LRH + LC
3	2018.11.14	285	100	0	NO	LLLH
4	2018.12.04	365	200	0	NO	LRH + LC
5	2018.12.14	240	20	0	NO	LRH + LC
6	2019.05.28	205	100	0	NO	LRH + LC
7	2019.07.23	285	250	0	NO	LWR + LC
8	2019.10.11	145	50	0	NO	LLLH
9	2019.10.17	235	50	0	NO	LLH + LC
10	2019.10.22	160	20	0	NO	LWR
11	2019.10.23	580	800	2	NO	LMH
12	2019.11.28	190	30	0	NO	LLLH
13	2019.11.28	235	150	0	NO	LWR

LWR: laparoscopic wedge resection; LRH: laparoscopic right hepatectomy; LLLH: laparoscopic left lateral hepatectomy; LLH: laparoscopic left hepatectomy; LMH: laparoscopic midlobe hepatectomy; LC: laparoscopic cholecystectomy.

### Postoperative conditions

All patients could walk and eat 1–3 days (mean: 1.2 days) after surgery. First flatus was found 1–3 days (mean: 1.7 days) after surgery. After surgery, drainage of the abdominal cavity was performed for 3–21 days (mean: 6.9 days) until no liquid was observed. The postoperative hospital stay lasted for 4–14 days (mean 7.2 days). Clinical symptoms of acute liver failure were observed in the second patient on the first postoperative day, and she was given treatment immediately. Fortunately, with positive treatment, her liver function gradually recovered and essentially returned to normal on the 7th postoperative day. On postoperative day 14, this patient met discharge criteria and was discharged with an abdominal drain, which was removed at a local hospital one week later. The seventh patient was also discharged with a drainage tube due to hydrops in the operation area. One week later, the effusion resolved spontaneously and then the drainage tube was removed in our hospital. All patients’ incisions healed well without incision-related complications. No mortality was reported during hospitalization. All patients were followed up. The average follow-up time was 15.7 months (9–30 months). During the follow-up period, no AE relapse was observed as revealed by US and/or CT in these patients (Table 3).

**Table 3:** Postoperative and follow-up information.

Case	Eating (*d*)	Emission (*d*)	Tube (*d*)		PHS (*d*)	Postoperative complications	Follow-up (months)		Recurrence
1	1	1		6	7	None		30	NO
2	1	3		21	14	Acute liver failure		27	NO
3	1	1		5	6	None		21	NO
4	3	3		9	9	None		20	NO
5	2	3		3	6	None		20	NO
6	1	1		4	5	None		15	NO
7	1	2		14	7	Hydrops		13	NO
8	1	1		3	7	None		10	NO
9	1	1		4	6	None		10	NO
10	1	1		6	8	None		10	NO
11	1	3		8	10	None		10	NO
12	1	1		3	4	None		9	NO
13	1	1		4	5	None		9	NO

PHS: postoperative hospital stay.

## Discussion

Hepatic alveolar echinococcosis, also known as hepatic multilocular echinococcosis, is a rare parasitic disease caused by the larval stage of *Echinococcus multilocularis* parasitising the human liver [16, 17]. Especially in the early and middle AE stages (P1N0M0/P2N0M0), it is particularly difficult to detect due to an absence of typical clinical symptoms. In addition, our team is not currently able to perform LH in most clinical AE patients because they are already in the advanced and/or terminal stage (P3 ~ 4NxMx) of the disease, in which lesions have invaded the important vessels of the hilum and/or patients have developed extrahepatic metastasis. These reasons explain the small number of cases in the present study.

Unlike other benign and malignant liver diseases, AE lesions have unique characteristics: large size, infiltrative growth, and hard texture. These characteristics may bring some challenges in terms of intraoperative exposure and intraoperative haemostasis. The second patient in our study developed an acute decrease in liver function after surgery because of uncontrollable massive haemorrhage caused by the peripheral small vessels that were constantly torn during separation from the firm lesion. This case shows that this minimally invasive surgical approach may involve some technical difficulties in practice.

Several published case reports on laparoscopic treatment for hepatic AE published successful results with this technique [6, 10, 18]. They did not propose the selection criteria for AE treatment by laparoscopy due to the limited number of patients.

To the best of our knowledge, this study reports the first successful attempt in the field of minimally invasive hepatectomy in more than 10 patients with hepatic AE. In our series, the results were promising, with a 100% survival rate and only one case having a complication above Clavien-Dindo grade III, which is acceptable. The advantages of this minimally invasive surgical approach were fully illustrated by the enhanced recovery after surgery of patients and short-term hospitalisation. Of note, it is important to develop specific surgical plans according to the actual situation of the patient. For example, although the fifth patient’s AE lesion was located in the right anterior lobe, we performed right half liver resection because of a CE cyst in the right posterior lobe simultaneously. In this way, the AE lesion and CE cyst could be removed simultaneously, effectively avoiding repeat surgery. In addition, the procedure for resecting a lesion in the middle liver lobe is more difficult because it is equivalent to the simultaneous completion of two hepatectomies. Therefore, we do not recommend attempting this approach for inexperienced surgical teams. Interestingly, all patients in this study did not take albendazole tablets regularly after surgery, but no relapse case was observed at 9–30 months follow-up.

**Figure 2:**
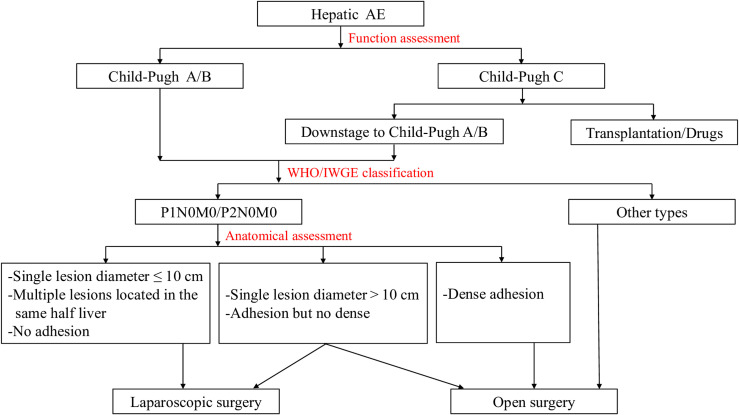
Treatment algorithms for hepatic alveolar echinococcosis.

With regard to the surgical indications of LH for AE treatment, we propose preliminary selective criteria (Fig. 2): (1) The patient’s cardiopulmonary function and general condition can tolerate laparoscopic surgery. (2) Preoperative liver function is Child-Pugh grade A or B. (3) According to the WHO-IWGE classification criteria, the AE stage should be P1N0M0/P2N0M0. (4) The single AE lesion diameter should be ≤10 cm. (5) Multiple AE lesions should all be located in the same half-liver. (6) The lesion does not adhere to the surrounding tissue. In addition to the above conditions, when the single lesion diameter exceeds 10 cm and/or the lesion is adherent to the surrounding tissue but no dense as armor, the surgical team should make the choice whether to perform LH according to the actual situation.

Although the results of our trial were encouraging, with only one patient experiencing a complication above Clavien-Dindo grade III and zero hospital mortality, we still consider that this study has some shortcomings. Firstly, the number of cases included was relatively small. Secondly, it is difficult to make an objective scientific evaluation of the superiority and inferiority of LH for AE treatment due to lacking direct comparison with classic open surgery. Therefore, further comparative studies with classical open hepatectomy are needed.

Finally, clinical practice guidelines have validated LH safety and feasibility, but we still considered that LH treatment for hepatic AE should be performed within institutions with experience in liver surgery.

In conclusion, the results of our preliminary study showed that LH is safe and feasible for radical treatment of hepatic AE. It can eliminate the whole AE lesion with a minimal wound. Strict patient selection and experienced surgical teams are key to successful surgery. The advantages of this technique for AE treatment need to be compared further with the classical open approach.

## Conflict of interest

There are no competing interests.
